# The South Carolina State Dental Association

**Published:** 1875-07

**Authors:** Theodore F. Chupein


### Proceedings of Societies.

### THE SOUTH CAROLINA STATE DENTAL ASSOCIATION.

The Fifth Annual Meeting of this Association was held on the 4th of May, in the city of Columbia, S. C.

The meeting was well attended and the discussions harmonious, spirited and interesting.

A Board of Dental Examiners, in accordance with the provisions of the bill recently passed by the Legislature of the state for regulating the practice of Dentistry in South Carolina, was elected, consisting of the following gentlemen: J. W. Norwood, Greenville, S. C.; J. B. Patrick, Charleston, S. C.; J. S. Thompson, Abbeville, S. C.; W. S. Brown, Charleston, S. C.; D. L. Boozer, Columbia, S. C.

Among the points of interest advanced at this meeting were the following:

Subcutaneous injections of ergotine and internal administration of same in form of pills for the suppression of violent and persistent hemorrhage after extraction. Application of actual cautery (white heat) by means of electricity for same purpose. Employment of electricity (white heat) for painless devitalizing of pulps.

Dr. Rice spoke against the employment of arsenic and said that he used with entire success a liniment consisting of ten grains acetate morphia to the ounce of carbolic acid which was almost a specific in his hands, curing the worst form of tooth-ache without devitalizing the nerve.

Flattering results had been experienced by many in the use of preparation of oxy-chloride of zinc for capping exposed or partially exposed pulps, some using it directly over the exposed parts, some using a covering of paper, kid, etc., and some using the white oxide of zinc next the exposed part and filling over this with the oxy-chloride zinc—flooding the the cavity before introducing the filling with carbolic acid or creosote, and using the rubber dam to protect against moisture.

A very favorable report was made by those who had used the "Modeling Compound" of S. S. White for taking impressions of the mouth—reports being that it was cleaner and pleasanter to both patient and operator, and taking a sharper impression than wax.

A like favorable report was made of "Parker and Teague's Impression Compound," granting to it all that was claimed for it by the inventors.

The following officers were elected for the ensuing year: G. F. T. Wright, Columbia, President; J. W. Norwood, Greenville, First Vice-President; B. H. Teague, Akin, Second Vice-President; J. T. Thompson, Abbeville, Corresponding Secretary; T. F. Chupein, Charleston, Recording Sect.; T, W. Boucheer, Cheraw, Treasurer.

The second Tuesday of June, 1876, was selected as the time, and Greenville the place for the next meeting.

After an address by the retiring President, the meeting adjourned to meet as above. THEODORE F. CHUPEIN,
Recording Secretary.

